# Genetics and clinical phenotypes in common variable immunodeficiency

**DOI:** 10.3389/fgene.2023.1272912

**Published:** 2024-01-11

**Authors:** Charlotte Cunningham-Rundles, Jean-Laurent Casanova, Bertrand Boisson

**Affiliations:** ^1^ Department of Medicine, Icahn School of Medicine at Mount Sinai, New York, NY, United States; ^2^ Department of Pediatrics, Icahn School of Medicine at Mount Sinai, New York, NY, United States; ^3^ Division of Clinical Immunology, Departments of Medicine and Pediatrics, Icahn School of Medicine at Mount Sinai, New York, NY, United States; ^4^ St. Giles Laboratory of Human Genetics of Infectious Diseases, Rockefeller Branch, The Rockefeller University, New York, NY, United States; ^5^ Laboratory of Human Genetics of Infectious Diseases, Necker Branch, INSERM, Necker Hospital for Sick Children, Paris, France; ^6^ Paris Cité Université, Imagine Institute, Paris, France; ^7^ Department of Pediatrics, Necker Hospital for Sick Children, Paris, France; ^8^ Howard Hughes Medical Institute, New York, NY, United States

**Keywords:** common variable immunodeficiency, genetics, autoimmunity, lung disease, granulomatous disease, cancer, lymphoma

## Abstract

Common variable immunodeficiency (CVID) is one of the most common symptomatic groups of inborn errors of immunity. In addition to infections resulting from insufficient levels of immune globulins and antibodies, many patients develop inflammatory or autoimmune conditions, which are associated with increased mortality. This aspect of CVID has been the focus of many studies, and dissecting the clinical phenotypes of CVID, has had the goal of providing biomarkers to identify these subjects, potentially at the time of diagnosis. With the application of whole exome (WES) and whole genome analyses, an increasing number of monogenic causes of CVID have been elucidated. From the standpoint of the practicing physician, an important question is whether the clinical phenotype, particularly the occurrence of autoinflammation of autoimmunity, might suggest the likelihood of identifying a causative mutation, and if possible the gene most likely to underlie CVID. We addressed this question in a patient group of 405 subjects diagnosed with CVID from one medical center.

## Introduction

Common variable immunodefciency (CVID) is one of the more frequently encountered immune defects in clinical practice, with an estimated incidence of about 1 in 20,000. The diagnosis is made in a male or female patient with reduced serum levels of IgG, along with IgA, and/or IgM with documented defects of antibody production to both protein and carbohydrate antigens, and the exclusion of other causes of hypogammaglobulinemia such as physiologic immaturity, medications, malignancy, or protein losses (Bonilla et al., 2016; Registry, 2016; Odnoletkova et al., 2018; Seidel et al., 2019). While considered genetic defects, most newly diagnosed patients are between the ages of 20 and 40 years old. (Resnick et al., 2012a; Gathmann et al., 2014; Odnoletkova et al., 2018). One of the clinical features of the CVID syndrome that has emerged is that about half of these patients have infections as the central manifestation, which can be successfully treated or prevented with antibiotics and immunoglobulins. However, the others also have various apparently non-infectious, autoimmune, autoinflammatory, neoplastic and/or lymphoproliferative manifestations, often associated with systemic immune activation (Wehr et al., 2008; Resnick et al., 2012b; Cols et al., 2016; Smith and Cunningham-Rundles, 2021; Ho and Cunningham-Rundles, 2022). Patients in the second group often have autoimmune or inflammatory features as the initial presentation and primary clinical manifestation, with less obvious susceptibility to significant infectious diseases; these subjects also have increased morbidity and mortality as compared to those with the infection-only phenotype (Chapel et al., 2008; Resnick et al., 2012a). A number of studies have probed reasons for the striking heterogeneity of this CVID patient pool (Wehr et al., 2008; Chapel and Cunningham-Rundles, 2009; Resnick et al., 2012a). These studies have sought biomarkers to identify these subjects, preferably at the time of diagnosis (Ho and Cunningham-Rundles, 2022). Some of these markers include identification of subjects with loss of peripheral isotype switched memory B cells, increased CD21^low^ B cells (<10%), and/or reduced numbers of T cells, especially naïve CD4 T cells (Warnatz et al., 2002; Fevang et al., 2007; Sanchez-Ramon et al., 2008; Wehr et al., 2008; Malphettes et al., 2009; Mouillot et al., 2010). Other markers more recently defined include elevated markers of systemic immune activation: serum lipopolysaccharide binding protein (LBP), sCD14 (Barbosa et al., 2012; Litzman et al., 2012; Fraz et al., 2022) and more recently, serum zonulin and circulating bacterial DNA (Ho et al., 2021). These correlative biomarkers attempt to distinguish many patients with the infection-only clinical phenotype from those with more inflammatory complications, but they do not address the molecular mechanisms.

**FIGURE 1:**
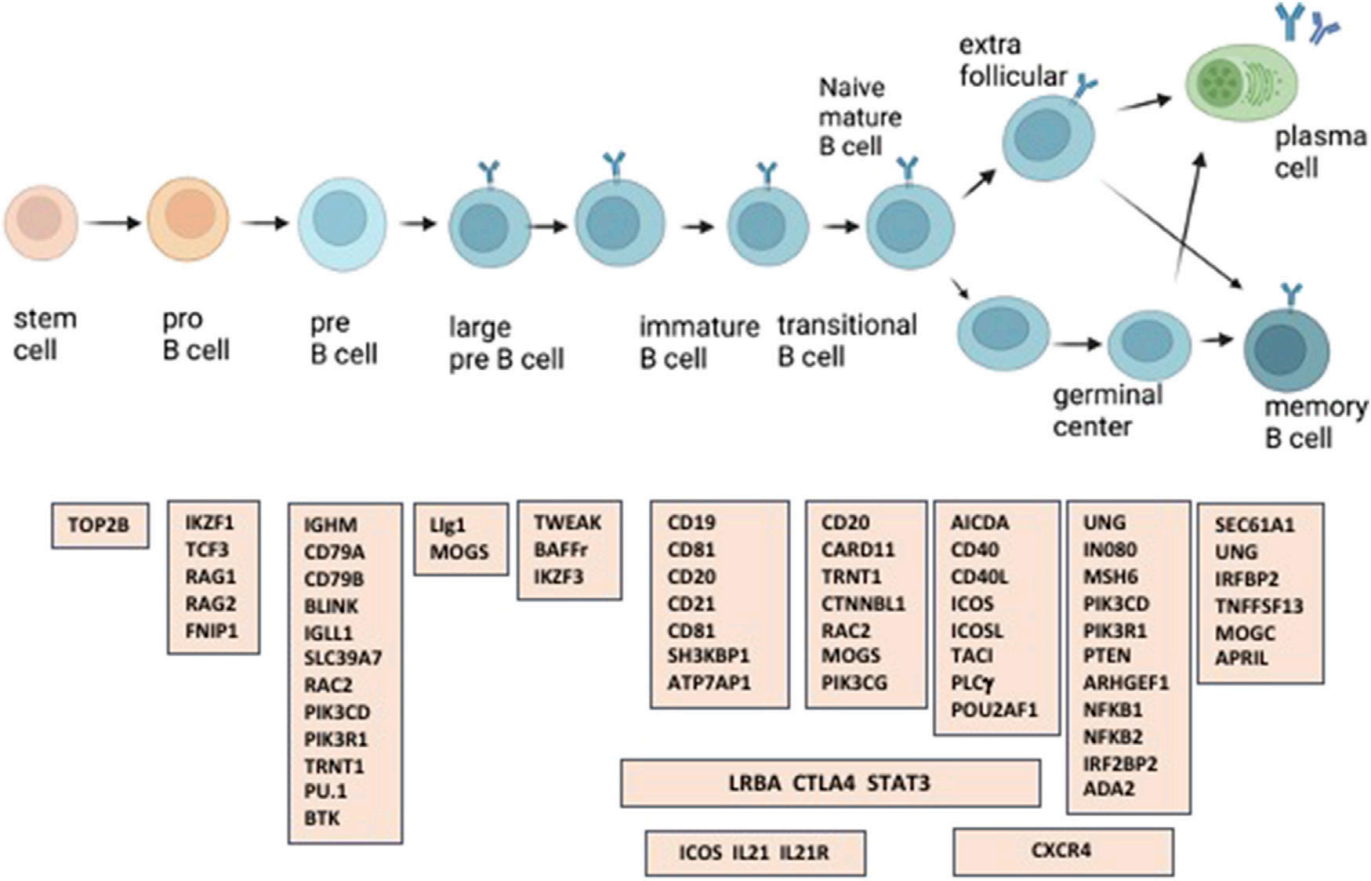
A number of the immune defects found in patients with CVID, are in genes involved in the generation and maturation of human B cells.

With the availability of whole exome (WES) and whole genome analyses (WGS), an increasing number of monogenic causes of the CVID phenotype have been elucidated, now accounting for about 25%–30% of subjects (Maffucci et al., 2016; Tuijnenburg et al., 2018; Abolhassani et al., 2020; Ramirez et al., 2021; Rojas-Restrepo et al., 2021). Several recent reports have described the results of genetic analyses of large CVID patient populations, with differences noted due to the location of the populations studied and ethnic background of the patients (Abolhassani et al., 2020; Rojas-Restrepo et al., 2021). The many genes identified in cohorts of subjects diagnosed with CVID, reflect the complex requirements of class switch recombination, B cell antigen signaling, activation, migration, long-term survival, and maturation and retention of antibody-secreting memory B cells into the plasma cell stage (Figure 1). From the standpoint of the practicing physician, an important question is whether the clinical phenotype suggests the possibility of identifying a causative mutation and if so, the gene(s) most likely to underlie the immune defect. Here we address this question in a large patient group from one medical center, encompassing an urban patient population on the East Coast of the United States.

## Methods

### Patient selection

Subjects were seen in the Immune Deficiency program at the Icahn School of Medicine at Mount Sinai. Patients were diagnosed with CVID using established criteria, including serum IgG and IgA and/or IgM deficiency with proven loss of antibody production (Bonilla et al., 2016; Picard et al., 2018; Seidel et al., 2019). Immunologic and clinical histories were collected from the clinical record and selected manifestations of inflammatory/autoimmune complications were recorded. For purposes of the current study, these sometimes partly overlapping medical conditions are autoimmunity, interstitial lung disease, granulomatous disease identified in one or more tissues, cancer, lymphoma, significant gastrointestinal disease, and previous splenectomy. Ethical permission for these studies was obtained from the Mount Sinai Institutional Review Board, and Informed consent was obtained from all individuals and/or their legal guardians.

#### Genetic evaluation

*Whole exome sequencing:* Genetic evaluation was done by whole exome sequencing (WES) as previously described (Maffucci et al., 2016; Picard et al., 2018; Maffucci et al., 2019). For this genomic DNA was extracted from peripheral blood mononuclear cells and sheared with a Covaris S2 Ultrasonicator. An adaptor-ligated library was prepared with the Paired-End Sample Prep kit V1 (Illumina). Exome capture was performed with the SureSelect Human All Exon kit (Agilent Technologies). Massively parallel sequencing was performed on a HiSeq 2,500 (Illumina), which generates 100-base reads. Sequences were aligned for variant calling and annotation with the human genome reference sequence (hg19 build) using BWA aligner (Li and Durbin, 2009). Downstream processing was performed with the Genome analysis toolkit (GATK) (McKenna et al., 2010), SAMtools (Li et al., 2009), and Picard Tools (http://picard.sourceforge.net/). A GATK UnifiedGenotyper and a GATK IndelGenotyperV2 were used to identify substitution and indel variant calls, respectively. Calls with a read coverage of ≤2x and a Phred-scaled single-nucleotide polymorphism (SNP) quality of ≤20 were filtered out. All variants were annotated with the GATK Genomic Annotator (Broad Institute). Heterozygous and homozygous variants were excluded if the allele frequencies in the general population were greater than 0.01% or 1.0%, respectively, in the Exome Aggregation Consortium database (ExAC, Broad Institute) and Genome Aggregation Database (gnomAD, Broad Institute) in respect with genetic model tested. This filtering strategy excluded polymorphic variants from consideration. Familial segregation was studied when samples were available. Other candidate mutations were confirmed by examining read alignment in the Integrated Genomics Viewer (IGV; Broad Institute). All confirmed mutations were subsequently analyzed using computational predictors of mutation severity including Sift (Ng and Henikoff, 2003), Polyphen 2 (Adzhubei et al., 2010). and Combined Annotation Dependent Depletion (CADD) (Kircher et al., 2014) and were compared with the gene-specific mutation significance cutoff (MSC) (Itan et al., 2016). Variants with CADD scores below the gene-specific MSC were excluded with the exception of *CXCR4*, *LIG1*, *LRBA* and *NFKB2*, 4 genes with a very high MSC (>32), but known to be causal of CVID. Confirmed variations were also screened through the Human Gene Mutation Database (Stenson et al., 2003) to identify published disease-associated variations. In a number of cases, the variants selected were tested for pathogenicity, and if not, categorized (as likely benign, VUS - Variant of Uncertain Significance, or likely pathogenic.). The pathogenicity of all disease attributable gene variants was evaluated using the updated guideline for interpretation of molecular sequencing by the American College of Medical Genetics and Genomics (ACMG) considering the allele frequency, computational data, immunological/functional data, familial segregation and parental data and clinical phenotyping (Richards et al., 2015).

*Using a targeting panel of genes:* More recently, patient exomes were also examined for mutations in 429 genes associated with a primary immune deficiency disease (Invitae Diagnostics), when faster results were needed and insurance or other payment for this service was available. For inheritance questions, the targeted sequence method was used in particular as it has the Clinical Laboratory Improvement Amendments (CLIA) certification needed for clinical decision analysis. As the targeted panel contains only a defined panel of genes, the above WES method was also used on these same samples so that the data could be verified, and so that additional queries for new genes could be undertaken at a later date.

##### Statistics

For evaluating the significance of genetics as related to clinical observations, Graphpad Prism was used; a *p*-value of less than 0.05 was considered significant.

## Results

### Patients

The Mount Sinai cohort of 405 genetically-tested CVID patients included 26 cases in which a family history was noted (Table 1). The age range of patients was 5–77 years, with median age of 44 years; 187 were female and 218 were male. Of these subjects, most were of European descent, 22 were Hispanic, 12 were Black, and 4 were Asian.

**TABLE 1:** CVID subjects.

Parameters	N
Number	405
Males	218
Females	187
Median age, years (range)	44 (5–77)
Relatives with immune defects	26
Gene candidates identified (%)	128 (31%)

### Mutations identified

In this cohort, 125 of 405 subjects (31%) had mutations considered deleterious while the remainder (280) had no clear genetic abnormality identified. As reported previously, around 10% of our patients (42 subjects) have variants in the *TNFRSF13B/TACI* gene (Transmembrane Activator and CAML Interactor) most of these known to be functionally deleterious (Salzer and Grimbacher, 2021). These included compound heterozygous mutations in 5 subjects, and homozygous mutations in one subject*.* These variants would be considered associated but not causative of CVID. (Table 2). Sixteen other subjects had autosomal dominant (AD) *NFKB1* deficiency (Nuclear Factor Kappa B Subunit 1), and 7 others had *NFKB2* (Nuclear Factor Kappa B Subunit 2) defects*,* both viewed as causative of this immune defect (Chen et al., 2013; Tuijnenburg et al., 2018; Li et al., 2021). More than one subject had mutations in heterozygous genes previously found in subjects with a CVID phenotype: *IRF2BP2, CTLA4 and IKZF1* (in 6 subjects each)*, TCF3* (in 5 subjects)*, BACH2* (in 4 subjects)*,* and in *STAT3, and PIK3CD* (3 subjects for each). Three other subjects had autosomal recessive (AR) deficiency of *LRBA*. Four adult subjects with infections, autoimmunity and mild retardation had mutations in *KMT2D* (Lysine Methyltransferase 2), a gene associated with Kabuki syndrome. Two sisters and the son of one of them, with no warts and moderate neutropenia, but severe autoimmune thrombocytopenia and autoimmune hemolytic anemia, had frameshift mutations in *CXCR4* (C-X-C Motif Chemokine Receptor 4), a gene associated with WHIM syndrome (warts, hypogammaglobulinemia, infections, and myelokathexis) (Maffucci et al., 2016; Abolhassani et al., 2020). Note that as in other reports, genes identified with previously un-identified X-immune linked defects were also noted in this cohort, *BTK*, *CD40L, IKBKG* and *WAS* (Table 2). Complicating the genetics is that in 15 subjects studied, more than one heterozygous variant, predicted to be deleterious, was identified. This included 8 subjects with at least one *TACI* variants, but additional variants in other autosomal dominant or recessive genes were also noted, for example, in *TBX1, TCF3, IL10-RA, NFKB2*, *NBAS, RAG1, RAG2* or a DiGeorge chromosome 22q deletion (Table 3). Further information related to minor allele frequency, and predicted deleterious effects on the selected variants are included in Methods and Supplementary Table S1.

**TABLE 2:** Gene variants identified - 125 subjects (31% of the group).

Gene variants	Number	Name	Inheritance
*TNFRSF13B^a^*	42	Transmembrane Activator and CAML Interactor	AD
*NFKB1*	16	Nuclear Factor Kappa B Subunit 1	AD
*NFKB2*	7	Nuclear Factor Kappa B Subunit 2	AD
*IRF2BP2*	6	Interferon regulatory factor-2 binding **protein**	AD
*CTLA4*	6	Cytotoxic T-Lymphocyte Associated Protein 4	AD
*IKZF1*	6	IKAROS Family Zinc Finger 1	AD
*TCF3*	5	Transcription Factor 3	AD
*BACH2*	5	BTB Domain and CNC Homolog 2	AD
*KMT2D*	4	Lysine Methyltransferase 2	AD
*STAT3*	3	Signal Transducer And Activator Of Transcription 3	AD
*PIK3CD*	3	Phosphatidylinositol-4,5-Bisphosphate 3-Kinase Catalytic Subunit Delta	AD
*LRBA*	3, compd het	LPS Responsive Beige-Like Anchor Protein	AR
*CXCR4*	3	C-X-C Motif Chemokine Receptor 4	AD
DiGeorge 22q11 or *TBX1*	3	DiGeorge syndrome	AD
*WAS*	2	Wiskott Aldrich syndrome	XL
*RAG1/RAG2*	2	Recombination Activating Genes 1/2	AR
*AICDA*	1, homozygous	Activation induced cytidine deaminase	AR
*STXBP2*	1, compd het	Syntaxin Binding Protein 2	AR
*PMM2*	1, compd het	Phosphomannomutase	AR
*PIK3R1*	1	Phosphoinositide-3-Kinase Regulatory Subunit 1	AD/AR
*LIG4*	1 homozygous	DNA Ligase 4	AR
*JAK1*	1	Janus Kinase 1	AR
*IKBKG*	1	Regulatory gamma subunit of the IKB kinase (IKK)	XL
TBX1	1	T-box protein 1	AD
PMS2	1, compd het	PMS1 Homolog 2, Mismatch Repair System	AR
*FOXP3*	1	Fork-winged helix family	XL
*LIG1*	1, compd het	DNA Ligase 1	AR
*CIITA*	1, compd het	Master regulator of MCH class II gene transcription	AR
*BTK*	1	Bruton Tyrosine Kinase	XL
*ADA2*	1	Adenosine deaminase 2	AR
*CD40L*	1	CD40 Ligand	XL
*RTEL1*	1, compd het	regulator of telomere elongation helicase 1	AR

^a^ Includes compound heterozygous mutations in 4; homozygous mutations in one; 8 of these also had an additional deleterious mutation in *TBX1, TCF3, IL10RA, NFKB2*, *NBAS,* or a DiGeorge chromosome loss; AD , autosomal dominant; AR , autosomal recessive; XL = X linked.

**TABLE 3:** Subjects with more than one gene variant.

Subject	Variant		Other variants	
*1*	*BACH2*	p.Gly483Ser	*POLE*	c.2706 + 1G>T
*2*	*BACH2*	p.Glu797*	*IKZF1*	p.Asn350His
*3*	*CTLA4*	c.109 + 1G>A	*IRFBP2*	p.Gln97His
*4*	*JAK1*	p.Asn76Ser	*STAT3*	p.Val461Leu
*5*	*NFKB2*	p.Gly719Glu	*TACI*	p.Leu69Thr fs*12
*6*	*NFKB2*	splicing	*TACI*	p.Cys104Arg
*7*	*PIK3R1*	start_gained/start_gained	*DCLRE1C*	del exon 1–3
*8*	*TACI*	p. Ala181Glu	*TBX1*	p.Leu1007Profs*2
*9*	*TACI*	p.Ala181Glu	Di George	22q11.2 deletion
*10*	*TACI*	p.Glu236*	*PMM2*	p.Arg141His
*11*	*TCF3*	p.Asn554Ser	*DOCK8*	c.54-1G>T
*12*	*TCF3*	p.Ile562Val	*TACI*	p.Leu69fs/p. Cys104Arg
*13*	*TCF3*	p.Pro96Leu	*TACI*	p.Lys188del
*14*	*TACI*	p.Ala181Glu	*IL10RA*	p.Arg147Profs*4
			*NBAS*	Deletion (Exons 45–52) p.Ser712*)
			*TMPRSS1*5	
*15*	*RAG1*	p.Asp212Asn	*RAG2*	p.Asp400His
*16*	*RAG1*	p.Asn968Lys	*RAG2*	p.Met110Leu

### Genetics and clinical phenotypes

**FIGURE 2:**
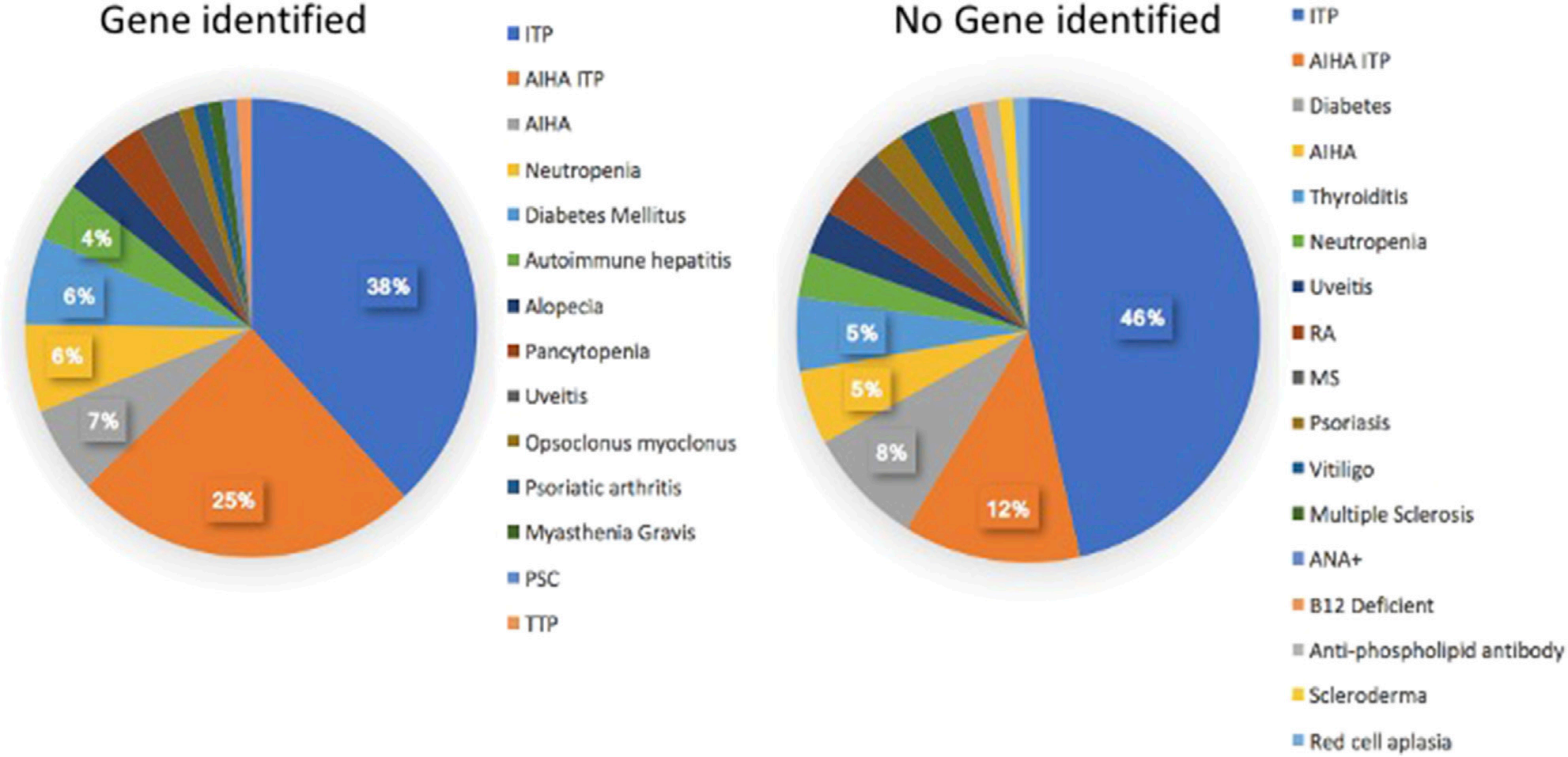
The kinds of autoimmune conditions found in patients with, and without identified genetic defects are similar.

We then examined if subjects with autoinflammatory, autoimmune, lymphoproliferative, neoplastic, granulomatous infiltrates, and/or gastrointestinal complications were more likely to have mutations in one or more of the genes identified in CVID, in contrast to others for whom a gene was not identified. Table 4 outlines the results for this cohort, dividing subjects according to whether or not a gene defect was identified in subjects with autoimmunity, significant pulmonary, gastrointestinal disease or liver disease, biopsy-demonstrated granulomatous disease, previous splenectomy, cancer, or lymphoma. Various forms of autoimmunity were noted in 151 subjects (37%) of the 405 group genetically tested, with no sex predominance. Of the 125 subjects with genes identified, 59 (47%) had autoimmunity, while of the larger group of 281 subjects with no gene noted, 32% had autoimmunity, suggesting some enrichment of this clinical feature in those with any known gene association; however these differences were not statistically significant. We also examined the types of autoimmunity in each group, in those with or without identified gene variants. Tables 5, 6 show these data. However, for both sets of patients, the most prominent autoimmune conditions were cytopenias, particularly thrombocytopenia, hemolytic anemia (or both, *i.e.*, Evan’s syndrome) or, in fewer numbers, neutropenia (Figure 2). The mutations found in those with autoimmunity are included in Supplementary Table S2. Note that of these, 14 had mutations in the *TACI* gene (2 with compound heterozygous variants, one with homozygosity) and 4 others were in association with additional deleterious variants). Five of the subjects with autoimmunity had mutations in *NFKB1*, 4 had variants in *CTLA4, IKZF1,* or in *STAT3,* and with other genes noted in other subjects.

**TABLE 4:** Percentage of complications in each group.

	Auto-immunity %	Pulmonary %	Gastro-intestinal %	Liver %	Granuloma %	Splenec-tomy %	Cancer %	Lymphoma %
With gene N= 125	47	31	16	17	17.6	13.6	8	6.5
no gene N= 280	32	17.5	18	9	7	6	4.3	7.5

**TABLE 5:** Autoimmunity: Gene identified.

N = 125			
Condition		Number	%
ITP		25	37
AIHA ITP		16	24
AIHA		4	6
Neutropenia		4	6
Diabetes Mellitus		4	6
Autoimmune hepatitis		3	4
Alopecia		2	3
Pancytopenia		2	3
Uveitis		2	3
Opsoclonus myoclonus		1	1
Psoriatic arthritis		1	1
Myasthenia Gravis		1	1
TTP		1	1
PSC		1	1

**TABLE 6:** Autoimmunity: No gene identified.

N = 280		
Condition	Number	%
ITP	42	45
AIHA ITP	11	12
Diabetes	7	8
AIHA	5	5
Thyroiditis	4	5
Neutropenia	3	3
Uveitis	3	3
RA	3	3
Psoriasis	2	2
Vitiligo	2	2
Multiple Sclerosis	2	2
ANA+	1	1
B12 Deficient	1	1
Anti-phospho-lipid antibody	1	1
Scleroderma	1	1
Red cell aplasia	1	1

AIHA , autoimmune hemolytic anemia; ITP , immune thrombocytopenia; TTP, thrombotic thrombocytopenic purpura; PSC , primary sclerosing cholangitis; ANA, antinuclear antibody; RA , rheumatoid arthritis; PSC , primary sclerosing cholangitis.

Significant pulmonary disease (interstitial lung disease, numerous nodules, and/or known granulomatous disease or respiratory failure) was observed in 39 subjects. Of these (31%) had an identified gene defect, as compared to 17.5% of those with no gene noted, suggesting a slight but not significant enrichment of significant respiratory disease in those with an identified gene, *p* = 0.053. Of those with significant lung disease, 12 subjects had a *TACI* variant, 4 had *NFKB2,* 4 had *NFKB1* and 3 others had *PI3KCD* variants. Mutations in the genes, BACH2, *KMT2D, LRBA, LIG4, STAT3 FOXP3* and *CASP8* were identified in other patients as outlined (Supplementary Table S3). Thirteen of these subjects had been diagnosed as having granulomatous lymphocytic infiltrates in the lungs (GLILD) (Bates et al., 2004). Overall, the tissue diagnosis of granulomatous disease (in lymph nodes, lung skin, or liver) was noted in 22 (17.7%) of the subjects with defects in known genes (B*ACH2, CTLA4, KMT2D, LRBA, PIK3R1, STAT3, TACI, TCF3 or WAS.*) This was significantly different from the 20 other subjects (7.5%) with granulomatous infiltrations) with no known gene defect, (*p* = 0.046).

The diagnosis of cancer or lymphoma was made in 18 patients (14.5%) with an identified gene defect, including 9 lymphoid malignancies and 6 other cancers, with the genetic changes noted for each (Table 7). Cancer or lymphoma was also diagnosed in 32 other subjects (11%) with no known genetic variants (not significantly different.) Gastrointestinal and/or liver disease were noted in similar proportions in each group, with the genes noted in Supplementary Table S4. Splenectomy, usually done for treatment of cytopenias, had been performed in 34 patients overall, with 17 (13.7%) of these in subjects with known genetic variants, as opposed to 17 others (6%) with no known genetic association (not significantly different.) Of the splenectomized subjects, 7 had TACI gene variants; others included *NFKB1* in 4, *LRBA, TCF3, STAT3, NFKB2, CTLA4* and *RAG1/RAG2*.

**TABLE 7:** Gene defects in cancers and lymphoma.

Gene	Consequence	Cancer	Lymphoma/Leukemia
*BTK*	p.Tyr418His	Esophagus	-
CD40L	indel-frameshift	Bladder	-
*IKZF1*	p.Ser385*		T cell leukemia
*LRBA*	p.Ile2232Thr/p.Ala892Thr	Mouth	-
*NFKB2*	p.His98Asn	-	Gastric Maltoma
*TACI*	p.Cys104Arg		
*NFKB2*	p.Gly719Glu	-	Gastric Maltoma
*TACI*	p.Leu69fs		
*PI3KCD*	p.Glu1021Lys	-	Lymphoma
*PI3KCD*	p.Glu1021Lys	Ovary	-
*PIK3R1*	start gained/start gained		MALT Lymphoma
*DCLRE1C*	del exon 1–3		
*RAB27A*.	del exon 2	Gall Bladder	
*PMS2*	p.Ile18Val/p.Arg563Leu		
*TACI*	p.Cys104Arg		Plasmablastic Lymphoma
*TACI*	p.Cys104Arg	Rectal	-
*TACI*	p.Ala181Glu	-	MALT Lymphoma
*TCF3*	p.Ile562Val	-	Lymphoma
*TACI*	p.Leu69fs/Cys104Arg		
*TACI*/	p.Ala181Glu		Lymphoma
*TMPRSS15*	p.Ser712*		
*NBAS*	deletion exons 45–52		
*IL10RA*	p.Arg147Pro fs*4		

We also considered if those with genetic variants might have specific types of unusual infections. However, a history of infections with *Herpes Zoster*, *Candida* sp, Giardia, *Clostridia difficile*, *Helicobacter pylori*, Norovirus, *Campylobacter*, *Herpes simplex*, or more unusual infections with atypical mycobacteria, *mycoplasma*, histoplasmosis, or cryptococcus, were seen in subjects with and without genetic variants.

## Discussion

**FIGURE 3:**
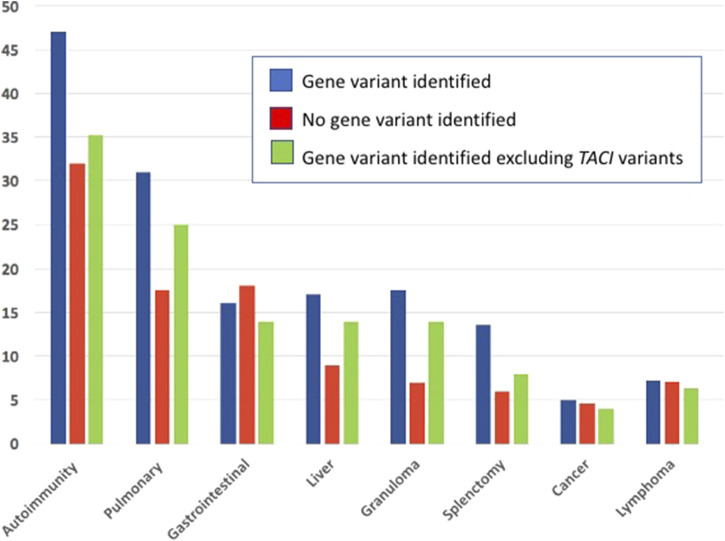
The medical complications noted in subjects with identified genetic defects, those with no known gene defects and subjects with a gene variant, but minus those with defects in the TNFRSF13B TACI receptor.

A number of previous studies have outlined the clinical phenotypes of large patient groups with antibody deficiency who have mutations in selected genes, including the *TACI* gene (Salzer et al., 2005; Zhang et al., 2007; Salzer et al., 2009), *CTLA4* (Schwab et al., 2018), *NFKB1* (Lorenzini et al., 2020), *NFKB2* (Klemann et al., 2019), *STAT3* (Fabre et al., 2019), *PI3KCD* (Jamee et al., 2020), or *LRBA* (Habibi et al., 2019). These studies describe the infectious, autoimmune and inflammatory characteristics of patients with these specific inborn errors of immunity. Here, the genetic analyses of CVID patients from one large cohort were examined to determine if the clinical complications might lead the physician to suspect a genetic defect in one or more of the previously established causal genes. In the current cohort of 405 subjects, 125 (31%) had an identifiable causative or associated genetic variant, similar to other investigated cohorts (Abolhassani et al., 2020; Rojas-Restrepo et al., 2021), however leaving the majority of patients without a known genetic cause. Autoimmunity was one of the commonest conditions for this group of patients with 37% of the patients having one or more of these conditions. Of these, 47% of them carried a predicted deleterious variant, while for those with no gene noted, 32% had autoimmunity. The autoimmune conditions noted were similar for each group, with cytopenias being the most prevalent manifestation, resulting in splenectomy in a number of subjects. While patients with mutations in *CTLA4, IKZF1, STAT3* and *LRBA* were in the autoimmune group, the largest number had variants in the *TACI* gene. While variants in the *TACI* gene are not thought to be disease causing, they are commonly associated with autoimmunity in CVID (Salzer et al., 2005; Zhang et al., 2007), possibly explaining this enrichment. If subjects with a TACI variant are excluded, 35% of subjects with a known gene were noted to have autoimmunity, more similar to those for whom no gene was identified (at 32%) as illustrated (Figure 3) The autoimmune cytopenia (ITP or AIHA) resulted in splenectomy in 34 patients, and 7 of the 17 with a known gene, had a *TACI* variant. Significant respiratory disease was also more common in those subjects with a known gene defect (at 31%). Excluding the 8 subjects with a *TACI* variant, 25% of these subjects had this complication, more similar to those with no known genetic background (17.5%). Granulomatous disease was overall, significantly enriched in those with genes identified. In this group, 17.6% had this complication if they had *TACI* variants; if these are excluded, the percentage was 14%, as opposed to 7% of those with no noted genes. Other complications, such as gastrointestinal, substantial liver disease or cancer appeared in subjects with and without identified genetic defects in similar numbers (Figure 3).

As the data presented here was gathered over a decade, a question that might arise if the DNA samples tested over time, using WES or the targeted panel, would lead to different results. We did not find this difficulty, but using the two methods led to confirmation. In addition, the accumulation of genetic data obtained by WES on all samples, also allowed for continued surveying for newly reported mutational differences. This allowed for updating as new genes contributing to the CVID phenotype were identified, and allowed all samples to be examined by the same parameters. The targeting panel was particularly useful for rapid analysis or inheritance questions, but even in these cases, WES was still performed. Limitations of this study include the fairly narrow demographic background of the subjects referred, a known reason for genetic differences in any CVID patient population (Abolhassani et al., 2020; Rojas-Restrepo et al., 2021), and the possibility of incomplete clinical information as these data were collected over varying periods of time. A further limitation is that confirmation of the pathogenicity of the identified variants depends on previous publications, *in vitro* assays where available, and the genetic methods used to assign the likelihood of a variant of dexterous variant exerting a deleterious change.

Overall, we conclude that CVID subjects with currently identifiable gene variants, either associated with or causative of this immune defect, appear to have an increased numbers of autoimmune manifestations, more significant respiratory disease and granulomatous changes in pathology; some of these differences can be attributed to co-existence of TACI variants as a genetic modifier. However, numerous other patients with no genetic basis yet discovered, have similar medical histories. It is possible that aside from genetics, these different clinical manifestations result from metabolic, environmental factors or epigenetic causes (Del Pino-Molina et al., 2019; Jorgensen et al., 2019; Macpherson et al., 2019; Ho et al., 2021; Rodriguez-Ubreva et al., 2022; Macpherson et al., 2023). While the majority of patients who carry the “CVID” diagnosis do not yet have a clarified molecular cause, the genetic discoveries in antibody defects continue to reveal the complex immunologic pathways needed to initiate and sustain normal B cell development and the long-term maintenance of B cell memory. With further exploration, more digenic or even polygenic causes of CVID are likely to be dissected, considering the intersecting immunologic pathways.

## Data Availability

The datasets presented in this study are available upon request.
