# Supplementary material for: Genetics and clinical phenotypes in common variable immunodeficiency
Source: Front Genet. 2024 Jan 11;14:1272912. doi: 10.3389/fgene.2023.1272912 (PMC10808799; doi:10.3389/fgene.2023.1272912)
Supplement: Supplementary file 1 [file DataSheet1.PDF]

Supplementary Table 1. Variants Identified

Gene	AA Change	Function	Zygo	gnomAD Gen.AF	gnomAD Ex.AF	Sift Pred.	Polyphen HVAR Pred.	CADD	MSC_99 %_Pred.	GDI	Consequence
AICDA	gene lost	homozygous loss	hom								Pathogenic
BACH2	p.Gly415Glu	missense	het	$2.4\ 10^{-3}$	$2.4\ 10^{-3}$	D	B	16.26	HIGH	2.54	VUS
BACH2	p.Gly483Ser	missense	het	$3.8\ 10^{-4}$	$1.4\ 10^{-4}$	T	B	1.49	LOW	2.54	VUS
BACH2	p.Arg666Lys	missense	het			D	P	33	HIGH	2.54	VUS
BACH2	p.Glu297Lys	missense	het			D	D	25.3	HIGH	2.54	VUS
BACH2	p.Arg576Gln	missense	het	$3.2\ 10^{-5}$	$1.6\ 10^{-5}$	T	D	24.7	HIGH	2.54	VUS
BACH2	p.Glu797*	stop-gained	het					42	HIGH	2.54	Pathogenic
BTK	p.Tyr418His	missense	hom	$3.2\ 10^{-4}$	$2.2\ 10^{-4}$	T	B	13.54	HIGH	0.42	VUS
CARD11	p.Ser212Arg	missense	het			T	P	23.6	HIGH	2.87	Likely pathogenic
CASP8	p.Gln448*	stop-gained	het		$1.6\ 10^{-5}$			35	HIGH	2.11	Pathogenic
CD40LG	p.Ser214fs	indel-frameshift	hom					25.9	HIGH	1.34	Likely pathogenic
CECR1	p.Gly47Arg	missense	het	$9.6\ 10^{-5}$	$1.1\ 10^{-4}$	D,	D	26.4	HIGH	6.29	Likely pathogenic
CIITA	p.Arg795Gln	missense	het	$6.4\ 10^{-5}$	$8.8\ 10^{-5}$	D	B	15.09	LOW	4.21	VUS
CIITA	p.Arg795Gln	missense	het	$6.4\ 10^{-5}$	$8.8\ 10^{-5}$	D	B	15.09	LOW	4.21	VUS
CTLA4	c.109+1G>A (Splice donor)	splice donor	het					23.7	HIGH	0.29	Likely pathogenic
CTLA4	p.Pro21fs	indel-frameshift	het					19.5	HIGH	0.29	Pathogenic
CTLA4	p.Pro136Ala	missense	het			D	D	23.8	HIGH	0.29	Likely pathogenic
CTLA4	p.Pro156Leu	missense	het		$8\ 10^{-6}$	T	D	33	HIGH	0.29	No
CTLA4	0	essential_splicing	het					25.1	HIGH	0.29	Pathogenic
CTLA4	p.Leu28fs	indel-frameshift	het					11.21	HIGH	0.29	Pathogenic
CXCR4	p.Val324fs	indel-frameshift	het					35	HIGH	0.29	Pathogenic
CXCR4	p.Val324fs	indel-frameshift	het					35	HIGH	0.29	Pathogenic
DCLRE1C	Deletion (Exons 1-3),	Deletion	het								Pathogenic
Chr 22 del	Chr 22 del	deletion	het								Pathogenic
DOCK8	0	essential_splicing	het	$2.2\ 10^{-4}$	$2.6\ 10^{-4}$			25.3	HIGH	18.29	VUS
FOXP3	p.Gly53Arg	missense	hom		$8.8\ 10^{-6}$	D	D	13.33	HIGH	1.23	VUS
IKBKG	p.Pro116Leu	missense	hom		$5.8\ 10^{-5}$	D	B	18.82	HIGH	0.09	VUS

IKZF1	p.Ser385*	stop-gained	het					35	HIGH	0.71	Pathogenic
IKZF1	p.Arg162Trp	missense	het			D	D	35	HIGH	0.71	Pathogenic
IKZF1	p.Arg184Gln	missense	het		$4.1\ 10^{-6}$	T	D	27.9	HIGH	0.71	Pathogenic
IKZF1	p.Gly242_Lys244delinsGlu	indel-inframe	het					34	HIGH	0.71	Pathogenic
IKZF1	p.Arg184Trp	missense	het			D	D	32	HIGH	0.71	Pathogenic
IKZF1	p.Arg162Trp	missense	het			D	D	35	HIGH	0.71	Pathogenic
IKZF1	p.Asn350His	missense	het								VUS
IL7R	p.Met1?	start-lost	het		$2.4\ 10^{-5}$	D	D	26.7	HIGH	2.87	VUS
IL10RA	p.Gln97His	Frameshift	het								unclear
IRF2BP2	p.Thr219Pro	missense	het			T	B	0	LOW	4.9	VUS
IRF2BP2	p.Pro127Ser	missense	het			D	B	11.58	HIGH	4.9	VUS
IRF2BP2	p.Ile389Ser	missense	het		$1.6\ 10^{-5}$	D	B	25	HIGH	4.9	VUS
IRF2BP2	p.Gln97His	missense	het								
IRF2BP2	p.Met192Val	missense	het			T	B	0	LOW	4.9	VUS
IRF2BP2	p.Pro238Thr	missense	het	$9.6\ 10^{-5}$	$2.8\ 10^{-3}$	D	B	11.93	HIGH	4.9	VUS
IRF2BP2	p.Ser283Arg	missense	het			T	B	2.08	LOW	4.9	VUS
IRF2BP2	p.Ile429Met	missense	het			T	D	12.77	HIGH	4.9	VUS
IRF2BP2	p.Gly436Glu	missense	het			D	D	27.5	HIGH	4.9	VUS
IRF2BP2	p.Pro118Ser	missense	het	$8.6\ 10^{-3}$	$2.3\ 10^{-3}$		B	10.56	HIGH	4.9	VUS
JAK1	p.Asn76Ser	missense	het	$3.2\ 10^{-5}$	$8.0\ 10^{-6}$	T	D	22.6	HIGH	1.69	Pathogenic
KMT2D	p.Gln4081*	stop-gained	het		$5.7\ 10^{-6}$			42	HIGH	6.74	Likely pathogenic
KMT2D	p.Arg5048Cys	missense	het			D	D	23.7	HIGH	6.74	Pathogenic
KMT2D	p.Cys1471Trp	missense	het			D	D	24.6	HIGH	6.74	Pathogenic
LIG1	p.Arg641Leu	missense	het	$3.2\ 10^{-5}$	$1.6\ 10^{-5}$	D	D	35	HIGH	4.47	Pathogenic
LIG1	p.Thr415fs	indel-frameshift	het	$3.2\ 10^{-5}$	$2.8\ 10^{-5}$			33	HIGH	4.47	Pathogenic
LIG4	p.Arg278His	missense	hom		$1.2\ 10^{-5}$	D	D	28.3	HIGH	8.69	Pathogenic
LRBA	p.K175*	stop-gained	het								Pathogenic
LRBA	p.T1597Rfs	Frameshift	het								Pathogenic
LRBA	p.Met467Val	missense	het	$2.7\ 10^{-3}$	$2.1\ 10^{-3}$	D	B	19	LOW	13.95	VUS
LRBA	p.Ala2784Gly	missense	het			T	D	25.7	LOW	13.95	VUS
LRBA	p.Ile2232Thr	missense	het	$6.7\ 10^{-4}$	$1.7\ 10^{-4}$	D	P	27.9	LOW	13.95	VUS
LRBA	p.Ala892Thr	missense	het	$3.3\ 10^{-3}$	$1.2\ 10^{-3}$	T	B	24.4	LOW	13.95	VUS

NBAS	deletion exons 45-52		het								unclear
NFKB1	p.Gln99*	stop-gained	het					38	HIGH	1.96	Pathogenic
NFKB1	p.Tyr319*	stop-gained	het					36	HIGH	1.96	Pathogenic
NFKB1	p.Lys117fs	indel-frameshift	het					23.2	HIGH	1.96	Pathogenic
NFKB1	0	essential_splicing	het					24	HIGH	1.96	Likely pathogenic
NFKB1	0	essential_splicing	het		$4.0\ 10^{-6}$			10.78	HIGH	1.96	Pathogenic
NFKB1	p.Gln199*	stop-gained	het					37	HIGH	1.96	Pathogenic
NFKB1	p.His110Arg	missense	het			D	P	25.7	HIGH	1.96	Pathogenic
NFKB1	0	essential_splicing	het		$4.0\ 10^{-6}$			10.78	HIGH	1.96	Pathogenic
NFKB1	0	essential_splicing	het					24.5	HIGH	1.96	Pathogenic
NFKB1	p.Thr869fs	indel-frameshift	het					35	HIGH	1.96	Isomorphic
NFKB1	p.Phe459fs	indel-frameshift	het					23.2	HIGH	1.96	Pathogenic
NFKB1	0	essential_splicing	het					24	HIGH	1.96	Pathogenic
NFKB1	p.Lys315_Thr316 delinsAsn	indel-inframe	het					20.3	HIGH	1.96	Likely pathogenic
NFKB2	p.His98Asn	missense	het			T	B	15.26	LOW	1.71	Pathogenic
NFKB2	p.Ser866Cys	missense	het			D	D	26.1	LOW	1.71	Pathogenic
NFKB2	p.Asp723Asn	missense	het	$1.1\ 10^{-3}$	$4.4\ 10^{-3}$	D	B	20.9	LOW	1.71	Isomorphic
NFKB2	p.Gly719Glu	missense	het		$8.1\ 10^{-6}$	T	B	0.59	LOW	1.71	Isomorphic
NKFB1	1210+1G>A spl donor	splice donor									Isomorphic (mRNA decay probable)
PIK3CD	p.Glu1021Lys	missense	het			D	D	31	HIGH	3.55	Pathogenic
PIK3R1	start_gained	start_gained	het					3.91	LOW	8.5	VUS
PIK3R1	start_gained	start_gained	het			D	P	25.1	HIGH	15.62	VUS
PLCG2	p.Asn571Ser	missense	het	$6.5\ 10^{-3}$	$6.7\ 10^{-3}$	D	B	22.9	HIGH	5.25	VUS
PLCG2	p.Tyr482His	missense	het	$3.4\ 10^{-3}$	$3.6\ 10^{-3}$	T	B	14.1	HIGH	5.25	VUS
PMM2	p.Arg141His	missense	het	$3.4\ 10^{-3}$	$4.0\ 10^{-3}$	D	B	34	HIGH	2.64	Likely pathogenic
PMS2	p.Ser248fs	Frame shift	het	$1.0\ 10^{-3}$			D	35	HIGH	5.54	Likely pathogenic
PMS2	p.Ser248fs	Frame shift	het	$1.0\ 10^{-3}$			B	14.71	HIGH	4.22	Likely pathogenic
POLE	0	essential_splicing	he		$4.0\ 10^{-6}$			23.6	HIGH	8.99	
POLE2	p.Glu169Val	missense	het		$2.1\ 10^{-5}$	D	D	33	HIGH	3.3	
POLE2	p.Glu169*	stop-gained	het					44	HIGH	3.3	

RAB27A	Deletion(Exon 2),	mIssense	het								Pathogenic
RAG1	p.Asn968Lys	missense	het			D	D	26.3	HIGH	8.76	VUS
RAG1	p.Asp212Asn	missense	het		$4.0\ 10^{-6}$	D	B	23.6	HIGH	8.76	VUS
RAG2	p.Met110Leu	missense	het			D	B	12.3	HIGH	3.54	VUS
RAG2	p.Asp400His	missense	het	$2.2\ 10^{-4}$	$5.4\ 10^{-4}$	D	D	25.3	HIGH	3.54	VUS
RTEL1	0	splicing	het		$4.2\ 10^{-6}$			4.71		6.8	Likely pathogenic
RTEL1	p.Cys1244fs	indel-frameshift	het		$8.1\ 10^{-6}$			24.8		6.8	Likely pathogenic
STAT3	p.Arg103Trp	missense	het			D	D	34	HIGH	1.52	VUS
STAT3	p.Arg246Gln	missense	het		$4.0\ 10^{-6}$	T	D	33	HIGH	1.52	Isomorphic
STAT3	p.Val461Leu	missense	het	$1.1\ 10^{-3}$	$6.7\ 10^{-3}$	T	B	23.1	HIGH	1.52	Isomorphic
STAT3	p.Phe313Leu	missense	het			T	D	16.66	HIGH	1.52	VUS
STXBP2	p.Pro345Leu	missense	het	$9.6\ 10^{-5}$	$6.4\ 10^{-5}$	D	D	23.4	HIGH	3.7	VUS
STXBP2	p.Cys169fs	indel-frameshift	het	$3.2\ 10^{-5}$	$8.2\ 10^{-5}$			33	HIGH	3.7	Likely pathogenic
TBX1	loss of coding gene	loss of coding gene	het								Pathogenic
TCF3	p.Pro96Leu	missense	het	$3.2\ 10^{-5}$	$2.0\ 10^{-4}$	D	B	24.2	HIGH	6.18	VUS
TCF3	p.Glu555Lys	missense	het			D	D	18.95	HIGH	6.18	Pathogenic
TCF3	p.Pro151Leu	missense	het	$3.2\ 10^{-5}$	$1.9\ 10^{-4}$	D	B	20.6	HIGH	6.18	VUS
TCF3	p.Ser455Ile	missense	het			D	B	23.7	HIGH	6.18	VUS
TCF3	p.Asn554Ser	missense	het		$8.0\ 10^{-6}$	D	D	25.1	HIGH	6.18	Pathogenic
TCF3	p.Ile562Val	missense	het	$3.2\ 10^{-5}$		D	D	17.03	HIGH	6.18	VUS
TET2	p.Glu1350*	stop-gained	het					54	HIGH	11.73	Likely pathogenic
TMPRSS15	p.Ser712*	stop-gained	het	$7.6\ 10^{-4}$	$6.3\ 10^{-4}$			48	HIGH	12.78	VUS
TNFRSF13B	p.Lys188del	indel-inframe	het	$9.6\ 10^{-5}$	$1.7\ 10^{-3}$			12.23	HIGH	5.15	VUS
TNFRSF13B	p.Cys104Arg	missense	het	$3.3\ 10^{-3}$	$3.5\ 10^{-3}$	D	D	25.9	HIGH	5.15	Pathogenic
TNFRSF13B	p.Ile87Asn	missense	het	$1.9\ 10^{-4}$	$4.6\ 10^{-4}$	D	D	25.2	HIGH	5.15	Pathogenic
TNFRSF13B	p.Glu236*	stop-gained	het	$3.2\ 10^{-5}$	$1.4\ 10^{-4}$			35	HIGH	5.15	VUS
TNFRSF13B	p.Ala181Glu	missense	het	$6.5\ 10^{-3}$	$5.3\ 10^{-3}$	D	P	22.8	HIGH	5.15	Pathogenic
TNFRSF13B	p.Lys188Met	missense	het	$1.4\ 10^{-2}$	$3.5\ 10^{-3}$	D	P	23.7	HIGH	5.15	Neutral
TNFRSF13B	p.Tyr164*	stop-gained	het	$6.4\ 10^{-5}$	$4.0\ 10^{-5}$			37	HIGH	5.15	Pathogenic
TNFRSF13B	p.Cys172Tyr	missense	het	$2.2\ 10^{-4}$	$1.8\ 10^{-4}$	D	P	24.8	HIGH	5.15	Pathogenic
TNFRSF13B	p.Leu69fs	indel-frameshift	het	$3.5\ 10^{-4}$	$4.1\ 10^{-4}$			28.6	HIGH	5.15	Pathogenic
TNFRSF13B	p.Leu171Arg	missense	het	$1.3\ 10^{-4}$	$1.0\ 10^{-4}$	D	D	25.4	HIGH	5.15	Pathogenic

TNFRSF13B	p.Ile87Asn	missense	het	1.9 10^{-4}	4.6 10^{-4}	D	D	25.2	HIGH	5.15	Pathogenic
TNFRSF13B	p.Ser194Tyr	missense	het	3.2 10^{-5}	1.2 10^{-5}		D	23.6	HIGH	5.15	VUS
WAS	p.Gln203Pro	missense	het			T	B	12.55	HIGH	1.14	VUS
WAS	p.Pro397del	indel-inframe	het					10.88	HIGH	1.14	VUS
WAS	p.Ile442Leu	missense	het			T	P	21.7	HIGH	1.14	VUS
